# Preprocedural cardiac computed tomography assessment of left atrial posterior wall morphology predicts atrial tachyarrhythmia recurrence after cryoballoon pulmonary vein isolation

**DOI:** 10.1016/j.hroo.2026.03.012

**Published:** 2026-03-20

**Authors:** Fuminori Odagiri, Takashi Tokano, Tetsuro Miyazaki, Takashi Iwasaki, Mariko Kobayashi, Ayane Kanai, Kenta Doi, Tomoko Sekine, Haruhiko Hara, Masato Tomaru, Takamasa Suzuki, Mari Sumiyoshi, Kai Ishii, Hiroshi Abe, Midori Kakihara, Masaaki Maki, Ryosuke Shimai, Hiroyuki Isogai, Dai Ozaki, Yuki Yasuda, Kiyoshi Takasu, Kazuhisa Takamura, Tohru Minamino

**Affiliations:** 1 Department of Cardiology, Juntendo University Urayasu Hospital, Urayasu-shi, Chiba, Japan; 2 Department of Radiology, Juntendo University Urayasu Hospital, Urayasu-shi, Chiba, Japan; 3 Department of Cardiovascular Biology and Medicine, Juntendo University Graduate School of Medicine, Bunkyo-ku, Tokyo, Japan

**Keywords:** Atrial fibrillation, Cryoballoon pulmonary vein isolation, Left atrial posterior wall, Cardiac computed tomography angiography, Atrial tachyarrhythmia recurrence

## Abstract

**Background:**

Pulmonary vein isolation (PVI) is the cornerstone of catheter ablation for atrial fibrillation (AF), yet its long-term efficacy remains limited. The left atrial posterior wall (LAPW) has emerged as an important arrhythmogenic substrate associated with recurrence after PVI.

**Objective:**

The purpose of this study was to determine whether LAPW morphology, quantified by preprocedural cardiac computed tomography angiography (CCTA), is associated with atrial tachyarrhythmia (ATA) recurrence after cryoballoon PVI (CB-PVI).

**Methods:**

We retrospectively analyzed 252 consecutive patients with AF (paroxysmal AF, n = 184 [73.0%]) undergoing first-time CB-PVI. LAPW morphology on CCTA was quantified as the left atrial roof line length, left atrial bottom line length, LAPW circumference (LAPC), and LAPW area. The primary end point was *ATA recurrence*, defined as any AF or atrial tachycardia episode ≥30 seconds after a 3-month blanking period.

**Results:**

During a median follow-up of 70 months (interquartile range 44.5–88.0 months), ATA recurred in 63 patients (25.0%). Patients with recurrence had larger LAPW dimensions. In multivariable Cox analysis, LAPC was significantly associated with recurrence (hazard ratio 1.079 per mm; 95% confidence interval 1.047–1.111 per mm; *P* < .001). Receiver operating characteristic curve analysis identified an LAPC threshold of ≥199.6 mm for recurrence (area under the curve 0.758; 95% confidence interval 0.698–0.818; *P* < .001).

**Conclusion:**

Preprocedural enlargement of the LAPW, particularly an increased LAPC, was significantly associated with ATA recurrence after CB-PVI. Quantitative assessment of LAPW morphology using routine preprocedural CCTA provides prognostic information that may complement conventional clinical and echocardiographic measures for recurrence risk stratification and individualized ablation strategies.

## Graphical abstract

**Figure:**
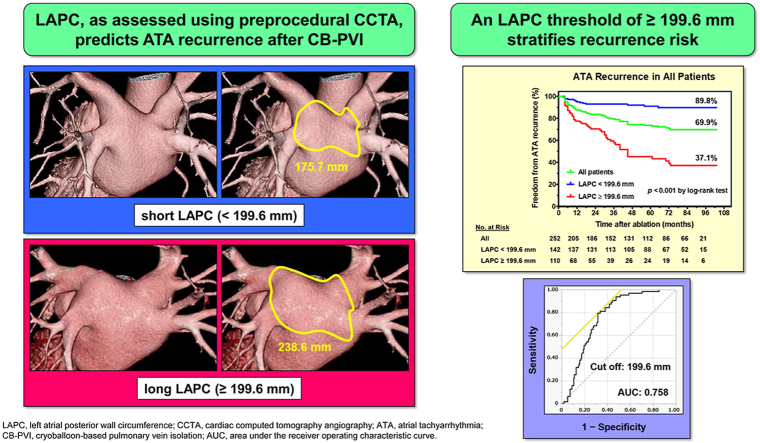

**Key Findings**

▪ Preprocedural cardiac computed tomography angiography (CCTA)–derived left atrial posterior wall morphology was associated with atrial tachyarrhythmia (ATA) recurrence after cryoballoon pulmonary vein isolation.
▪ Among CCTA-derived posterior wall parameters, left atrial posterior wall circumference (LAPC) emerged as the strongest regional imaging correlate of ATA recurrence after adjustment for conventional clinical and echocardiographic factors.
▪ An LAPC threshold of ≥199.6 mm provided effective risk stratification for ATA recurrence across atrial fibrillation subtypes.

## Introduction

Pulmonary vein isolation (PVI) remains the cornerstone of catheter ablation for atrial fibrillation (AF) and an essential component of contemporary AF management.^1^ Despite substantial advances in ablation technology and procedural techniques, the long-term efficacy of PVI alone remains suboptimal. Atrial tachyarrhythmia (ATA) recurrence has been reported in ∼40%–50% of patients with persistent AF (PerAF)^2^^,^^3^ and in 20%–30% of those with paroxysmal AF (PAF).^4^, ^5^, ^6^ Identifying patients at high risk for recurrence after PVI is crucial for optimizing ablation strategies and improving long-term rhythm outcomes. Although adjunctive ablation strategies beyond PVI remain under investigation,^7^ growing evidence supports individualized approaches to reduce ATA recurrence. The left atrial posterior wall (LAPW) has emerged as an important non–pulmonary vein (PV) substrate implicated in AF initiation and maintenance.^8^ Anatomical and electrical remodeling of the LAPW may contribute to arrhythmia persistence and recurrence after PVI, suggesting a potential role for LAPW-targeted ablation in selected patients. In addition, several clinical and echocardiographic factors, including left atrial (LA) size, AF subtype, and severity of mitral regurgitation (MR), have been identified as predictors of postablation recurrence.^9^ However, these markers provide limited insight into the structural characteristics of the atrial substrate. Noninvasive imaging modalities such as transthoracic echocardiography (TTE), cardiac magnetic resonance imaging, and cardiac computed tomography angiography (CCTA) are increasingly used to evaluate atrial remodeling and support individualized procedural planning.^10^ Among them, CCTA offers excellent spatial resolution, allowing accurate delineation of LA and PV anatomy and evaluation for intracardiac thrombus.^11^ Nevertheless, the prognostic significance of LAPW morphology derived from CCTA has not been fully established. Therefore, this study aimed to determine whether preprocedural CCTA–derived LAPW morphology is associated with ATA recurrence after cryoballoon PVI (CB-PVI). Clarifying the role of LAPW geometry may help inform risk stratification and individualized ablation strategies in patients with AF.

## Methods

### Study design and population

This retrospective, single-center, observational study included 252 consecutive Japanese adults (≥18 years) with AF who underwent first-time PVI using a CB catheter between June 2016 and December 2024. Eligible AF subtypes were classified as *PAF* (defined as AF terminating spontaneously or with intervention within 7 days), PerAF (lasting >7 days), and long-standing PerAF (LS-PerAF; continuous AF for >1 year). *Symptomatic AF* was defined as a patient-reported episode (eg, palpitations, dyspnea, or chest discomfort) confirmed by 12-lead electrocardiography (ECG) or 24-hour Holter monitoring. Among the study population, 184 patients (73.0%) had PAF while the remaining 68 patients (27.0%) had PerAF, including 5 patients (2.0% of the total) with LS-PerAF.

Exclusion criteria included the following: sustained AF >3 years; prior LA ablation; cerebral ischemic event, myocardial infarction, or unstable angina within 2 months; presence of intracardiac thrombus; left ventricular ejection fraction (LVEF) < 35%; LA diameter ≥ 60 mm; congenital heart disease; end-stage renal disease requiring dialysis; pregnancy; contraindication to general anesthesia or oral anticoagulant therapy; life expectancy < 1 year; follow-up duration < 3 months; or inability to provide written informed consent.

The study protocol adhered to the principles of the Declaration of Helsinki and was approved by the institutional ethics committee. Written informed consent was obtained from all participants.

### Objectives and end points

The primary objective was to determine whether preprocedural CCTA–derived anatomic and morphologic characteristics of the LA were associated with ATA recurrence after CB-PVI. The secondary objective was to identify clinical predictors of ATA recurrence in this cohort.

The primary end point was *ATA recurrence*, defined as any AF or atrial tachycardia (AT) episode ≥30 seconds after a 3-month blanking period (BP).

### Preprocedural management

Baseline demographic and clinical data were collected for all patients. TTE was performed within 6 months before catheter ablation to assess cardiac structure, including left ventricular function, LA size, and valvular abnormalities. Valvular heart disease severity was graded as mild, moderate, or severe based on color Doppler echocardiography. All examinations were performed by trained sonographers and interpreted by level III board-certified echocardiographers in accordance with established guidelines.^12^^,^^13^ All antiarrhythmic drugs (AADs) were discontinued for at least 5 elimination half-lives before the procedure. For amiodarone, discontinuation ≥ 1 month was recommended at the discretion of the treating cardiologist, considering pharmacokinetics and clinical status. Transesophageal echocardiography was performed routinely on the day before the procedure to exclude intracardiac thrombus. All patients received ≥3 weeks of uninterrupted oral anticoagulant therapy. Warfarin and dabigatran were continued without interruption. For patients receiving rivaroxaban, apixaban, or edoxaban, a temporary periprocedural switch to dabigatran was implemented per institutional protocol to standardize anticoagulation management. Preprocedural B-type natriuretic peptide levels were measured as part of standard evaluation.

### Preprocedural CCTA

All patients underwent CCTA within 1–4 weeks before catheter ablation to delineate LA and PV anatomy, evaluate for intracardiac thrombus, and enable image integration with the electroanatomic mapping system.

CCTA was performed on a 320-slice multidetector scanner (Aquilion ONE/INSIGHT Edition, Canon Medical Systems, Chiba, Chiba, Japan) using prospective ECG gating and 3-dimensional reconstruction. Iodinated contrast (Iomeron 350; iomeprol 350 mg I/mL) was administered at 50 mL via power injection (5 mL/s), followed by a 45-mL saline flush; the iodine delivery rate was standardized at 22 mg I/(kg·s). Acquisition parameters included detector collimation 320 × 0.5 mm, gantry rotation time 275 ms, and automatic exposure control. A tube potential of 100 kV was used in most patients; 120 kV was applied for body mass index > 30 kg/m^2^ or body weight > 90 kg. Images were reconstructed with a slice thickness of 0.5 mm and an increment of 0.25 mm using adaptive iterative dose reduction (AIDR 3D Enhanced, Standard mode; Canon Medical Systems Corporation, Otawara, Tochigi, Japan). ECG triggering targeted late ventricular systole (LA diastolic phase) with acquisition centered at 70%–80% of the RR interval. All scans were obtained during an inspiratory breath-hold using a prospective ECG-triggered protocol.

### CCTA measurements

**Figure 1:**
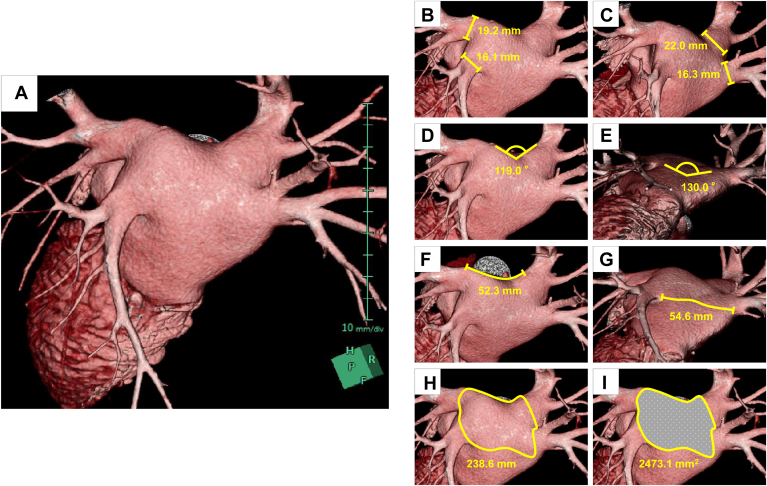
Representative preprocedural CCTA findings in a patient with PerAF who had recurrence of ATA after CB-PVI, demonstrating a long LAPC (≥199.6 mm). **A:** Reference axial slice. **B:** Ostial diameters of the LSPV and LIPV. **C:** Ostial diameters of the RSPV and RIPV. **D:** LA roof angle. **E:** LA bottom angle. **F:** LA roof line length. **G:** LA bottom line length. **H:** LAPC. **I:** LAPA. ATA = atrial tachyarrhythmia; CB-PVI = cryoballoon-based pulmonary vein isolation; CCTA = cardiac computed tomography angiography; LA = left atrial; LAPA = left atrial posterior wall area; LAPC = left atrial posterior wall circumference; LIPV = left inferior pulmonary vein; LSPV = left superior pulmonary vein; PerAF = persistent atrial fibrillation; RIPV = right inferior pulmonary vein; RSPV = right superior pulmonary vein.

**Figure 2:**
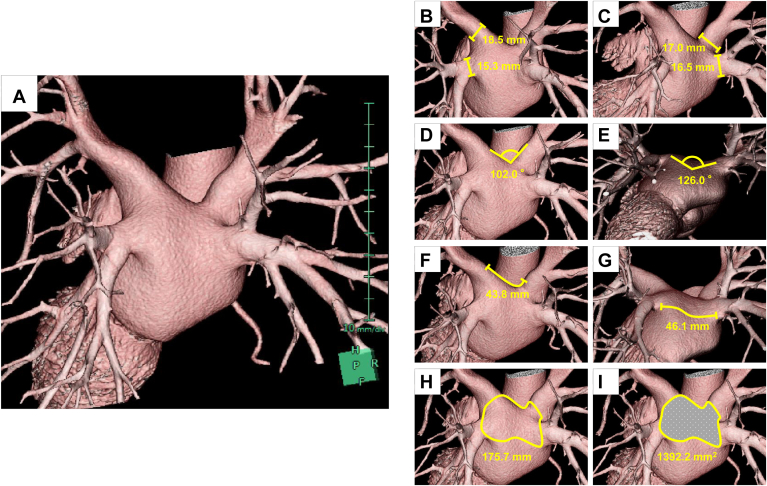
Representative preprocedural CCTA findings in a patient with PAF who had no recurrence of ATA after CB-PVI, demonstrating a short LAPC (<199.6 mm). **A:** Reference axial slice. **B:** Ostial diameters of the LSPV and LIPV. **C:** Ostial diameters of the RSPV and RIPV. **D:** LA roof angle. **E:** LA bottom angle. **F:** LA roofline length. **G:** LA bottom line length. **H:** LAPC. **I:** LAPA. ATA = atrial tachyarrhythmia; CB-PVI = cryoballoon-based pulmonary vein isolation; CCTA = cardiac computed tomography angiography; LA = left atrial; LAPA = left atrial posterior wall area; LAPC = left atrial posterior wall circumference; LIPV = left inferior pulmonary vein; LSPV = left superior pulmonary vein; PAF = paroxysmal atrial fibrillation; RIPV = right inferior pulmonary vein; RSPV = right superior pulmonary vein.

Morphologic assessment of the PVs, LAPW, and LA appendage (LAA) was performed preprocedurally on reconstructed CCTA data sets by visual inspection and manual caliper measurements (Figures 1 and 2). All CCTA measurements were performed by a study electrophysiologist (F.O.), who also served as 1 of the 2 operators for catheter ablation in this cohort. The *LA roof line length* was defined as the straight-line distance between the superior ostial hinge points of the right and left superior PVs on a standardized axial oblique plane orthogonal to the LAPW. The *LA bottom line length* was analogously defined between the inferior ostial hinge points of the right and left inferior PVs. The *LAPW circumference* (LAPC) was defined as the curvilinear perimeter of the LAPW, obtained by spline-tracing the atrial contour bounded superiorly by the roof line, inferiorly by the bottom line, and laterally by perpendicular lines through the PV ostial hinge points. This approach captures the anatomically irregular, nonrectangular geometry of the LAPW rather than assuming a rectangular shape, thereby distinguishing LAPC from conventional global LA size measures. LAPC was measured along the endocardial border on a standardized axial oblique plane using a spline-based tracing tool available in standard commercial CCTA workstation software. The LAPW area (LAPA) was measured as the planimetric area enclosed by these boundaries. CCTA-derived LAPW measurements (including LAPC and LAPA) were analyzed as absolute values and were not indexed to body surface area. Because LAPC represents the overall geometric extent of the LAPW and was hypothesized to best capture remodeling, reproducibility testing focused on LAPC. For interobserver agreement, 30 randomly selected patients (11.9% of the cohort) were reanalyzed by a second observer (K.I.), who was independent of the operators and blinded to clinical outcomes and index measurements; the sample size followed published recommendations.^14^^,^^15^

### Ablation procedure

All ablation procedures were performed under moderate sedation using a continuous dexmedetomidine infusion with supplemental bolus doses of pentazocine and thiopental as needed. Noninvasive ventilation via a nasal mask was maintained throughout the procedure. After vascular access, intravenous heparin (100 IU/kg body weight) was administered and a single transseptal puncture was performed using a radiofrequency (RF) needle (Baylis Medical, Montreal, Quebec, Canada) under fluoroscopic and intracardiac echocardiographic guidance (ViewFlex, Abbott, St. Paul, MN). After transseptal access, systemic anticoagulation was maintained with a target activated clotting time of ≥300 seconds. CB ablation was performed using either a second-generation (Arctic Front Advance, n = 130) or a fourth-generation (Arctic Front Advance PRO, n = 122) catheter (Medtronic, Minneapolis, MN), selected according to device availability. A 28-mm balloon was advanced into the LA via a 15-F steerable sheath (FlexCath Advance, Medtronic), and a circular inner-lumen mapping catheter (Achieve, Medtronic) was used to guide balloon positioning at each PV ostium. After confirming complete PV occlusion using selective venography, 1 or 2 CB applications (180–240 seconds each) were delivered per vein, following established dosing protocols.^16^^,^^17^ Luminal esophageal temperature was continuously monitored to prevent thermal injury, with applications immediately terminated if luminal esophageal temperature reached ≤18°C. During right PV ablation, phrenic nerve (PN) function was monitored by pacing from the superior vena cava or right subclavian vein with recording of compound motor action potentials. Freezing was aborted if compound motor action potential amplitude decreased by ≥30% or diaphragmatic movement diminished. Electrical cardioversion was performed when AF persisted after PVI or recurred during the procedure. *PVI success* was defined as bidirectional conduction block between the PVs and the LA, confirmed using the mapping catheter. Supplemental touch-up RF ablation (FlexAbility ablation catheter, Abbott, Chicago, IL) was applied only when complete isolation could not be achieved with the CB alone.

After PVI, ATA induction testing was performed using burst pacing from the right atrium and coronary sinus at cycle lengths from 300 ms down to the atrial refractory period (or 200 ms if not reached), sustained for 10 seconds, with or without isoproterenol infusion. Persistent induced ATAs (>5 minutes) were terminated by electrical cardioversion. All procedures were performed by 2 experienced electrophysiologists (F.O. and T.T.) with expertise in CB-PVI.

### Postprocedural management and follow-up

After ablation, all patients were continuously monitored with cardiac telemetry until hospital discharge, typically on postprocedural day 2. Preprocedural AADs were resumed at the discretion of the treating cardiologist. Patients were followed at 1 month after the procedure and subsequently every 2–3 months, or earlier if arrhythmic symptoms occurred. At each visit, a 12-lead ECG was recorded. In addition, 24-hour Holter monitoring was scheduled at 3, 6, and 12 months and annually thereafter to identify both symptomatic and asymptomatic arrhythmias. *ATA recurrence* was defined as any AF or AT episode ≥30 seconds after a 3-month BP.

### Statistical analysis

Continuous variables were expressed as mean ± standard deviation or as median with interquartile range (IQR), depending on distribution. Categorical variables were presented as count and percentage. Between-group comparisons were performed using the Student *t* test or the Mann-Whitney *U* test for continuous variables and the χ^2^ test or the Fisher exact test for categorical variables, as appropriate. Time-to-event analysis for ATA recurrence was performed using the Kaplan-Meier method, with intergroup differences assessed using the log-rank test. Univariable and multivariable Cox proportional hazards models were used to identify predictors of recurrence. All Cox regression analyses were performed using continuous variables; categorical cutoffs, including the LAPC threshold, were applied only for visualization and risk stratification in Kaplan-Meier analyses. All statistical analyses were performed using SPSS software (version 29.0, IBM Corporation, Armonk, NY) and JMP Pro (version 18.0, SAS Institute Inc., Cary, NC). A 2-sided *P* value <.05 was considered statistically significant. Interobserver agreement for CCTA-derived measurements was assessed using the intraclass correlation coefficient (ICC^1^^,^^2^), based on a 2-way random-effects model with absolute-agreement definition, according to published guidelines.^14^ ICC values >0.90 were considered excellent, 0.75–0.90 good, 0.50–0.75 moderate, and <0.50 poor. Although observers were not randomly selected, this model was chosen to evaluate the generalizability of the measurement method. Agreement was further analyzed using Bland-Altman plots, reporting mean bias and 95% limits of agreement.^18^

## Results

### Baseline characteristics

Baseline characteristics of the study population are summarized in Table 1. A total of 252 patients were included, of whom 63 (25.0%) experienced ATA recurrence (Rec group) and 189 (75.0%) did not (Non-rec group) during a median follow-up of 70.0 months (IQR 44.5–88.0 months). All patients completed follow-up without loss. The mean age of the entire cohort was 63.7 ± 11.9 years, with no significant difference between the Rec and Non-rec groups (62.6 ± 12.3 years vs 64.1 ± 11.8 years; *P* = .388). The proportion of male patients was similar (71.4% vs 74.6%; *P* = .629), and body size parameters, including height, weight, and body mass index, were comparable. The prevalence of comorbidities, including hypertension, diabetes mellitus, heart failure, coronary artery disease, chronic kidney disease, and prior stroke or transient ischemic attack, did not differ significantly between groups. However, non-PAF (PerAF or LS-PerAF) was more frequent in the Rec group (39.7% vs 22.8%; *P* = .009) whereas PAF predominated in the Non-rec group (77.2% vs 60.3%; *P* = .009). Symptomatic AF episodes were less common in patients with recurrence (65.1% vs 77.8%; *P* = .045). Stroke risk scores (CHADS_2_ and CHA_2_DS_2_-VASc) were low in both groups and showed no significant differences (CHADS_2_: 0.8 ± 0.9 vs 0.9 ± 1.0; *P* = .342; CHA_2_DS_2_-VASc: 1.6 ± 1.2 vs 1.7 ± 1.3; *P* = .384). Bleeding risk, as reflected by the HAS-BLED score, was also similar (1.1 ± 0.9 vs 1.2 ± 0.9; *P* = .643). The use of preprocedural medications, including renin-angiotensin system inhibitors, aldosterone antagonists, statins, β-blockers, and sodium-glucose cotransporter 2 inhibitors, did not differ between groups. Similarly, AAD use (class I agents, amiodarone, and bepridil) was comparable. In patients with non-PAF, pharmacologic restoration of sinus rhythm before ablation was achieved in 44.0% of the Rec group and 60.5% of the Non-rec group (*P* = .189). Preprocedural B-type natriuretic peptide levels tended to be higher in the Rec group, though not statistically significant (62.9 [32.8–113.2] pg/mL vs 33.1 [16.6–67.4] pg/mL; *P* = .102). TTE revealed a significantly larger LA diameter (41.9 ± 5.7 mm vs 38.8 ± 5.2 mm; *P* < .001) and LA volume index (LAVI) (39.5 ± 13.7 mL/m^2^ vs 32.9 ± 10.0 mL/m^2^; *P* < .001) in the Rec group. LVEF was similar (63.0% ± 10.0% vs 64.8% ± 8.3%; *P* = .203). Valvular heart disease (≥mild) tended to be more frequent in the Rec group (34.9% vs 22.8%; *P* = .056). Importantly, ≥moderate MR was significantly more common in patients with recurrence (15.9% vs 6.9%; *P* = .032).

**Table 1:** Baseline patient characteristics

Characteristic	Recurrence	Non-recurrence	*P*
No. of patients	63	189	
Age (y)	62.6 ± 12.3	64.1 ± 11.8	.388
Male sex	45 (71.4)	141 (74.6)	.629
Height (cm)	167.2 ± 9.6	166.1 ± 9.0	.421
Weight (kg)	68.9 ± 13.1	66.9 ± 13.2	.314
BMI (kg/m^2^)	24.7 ± 4.2	24.1 ± 3.6	.317
Comorbidities			
Congestive heart failure	5 (7.9)	16 (8.5)	.895
Hypertension	29 (46.0)	90 (47.6)	.827
Diabetes mellitus	5 (7.9)	29 (15.3)	.136
Previous stroke or TIA	1 (1.6)	5 (2.6)	.633
Coronary artery disease	5 (7.9)	8 (4.2)	.250
CKD (Chronic Kidney Disease Epidemiology Collaboration estimated glomerular filtration rate [CKD-EPI eGFR] < 60 mL/(min·1.73 m²))	1 (1.6)	8 (4.2)	.327
PAF	38 (60.3)	146 (77.2)	.009
PerAF	22 (34.9)	41 (21.7)	.036
LS-PerAF	3 (4.8)	2 (1.1)	.101
Symptomatic AF episode	41 (65.1)	147 (77.8)	.045
CHADS_2_ score	0.8 ± 0.9	0.9 ± 1.0	.342
CHA_2_DS_2_-VASc score	1.6 ± 1.2	1.7 ± 1.3	.384
HAS-BLED score	1.1 ± 0.9	1.2 ± 0.9	.643
Preprocedural medications			
ACE-I or ARB	21 (33.3)	57 (30.2)	.637
Aldosterone antagonist	0 (0)	3 (1.6)	.314
ARNI	0 (0)	2 (1.1)	.412
Statin	14 (22.2)	40 (21.1)	.859
β-Blockers	23 (3.7)	79 (41.8)	.459
SGLT2 inhibitors	0 (0)	5 (2.6)	.192
Preprocedural AAD therapy			
None	18 (28.6)	55 (29.1)	.936
Class Ⅰ	11 (17.5)	39 (20.6)	.584
Amiodarone	6 (9.5)	16 (8.5)	.797
Bepridil	28 (44.4)	79 (41.8)	.713
Pharmacologic restoration of SR before the procedure in non-PAF∗	11/25 (44.0)	26/43 (60.5)	.189
Pre-BNP (pg/mL)	62.9 (32.8–113.2)	33.1 (16.6–67.4)	.102
TTE parameters			
LVEF (%)	63.0 ± 10.0	64.8 ± 8.3	.203
LA diameter (mm)	41.9 ± 5.7	38.8 ± 5.2	<.001
LA volume index (mL/m^2^)	39.5 ± 13.7	32.9 ± 10.0	<.001
VHD (≥mild)	22 (34.9)	43 (22.8)	.056
MR ≥mild	15 (23.8)	27 (14.3)	.079
MR ≥moderate	10 (15.9)	13 (6.9)	.032

Values are presented as mean ± standard deviation, median (interquartile range), or n (%).

AAD = antiarrhythmic drug; ACE-I = angiotensin-converting-enzyme inhibitor; ARB = angiotensin receptor blocker; ARNI = angiotensin receptor-neprilysin inhibitor; BMI = body mass index; BNP = brain natriuretic peptide; CKD = chronic kidney disease; LA = left atrial; LS-PerAF = long-standing persistent atrial fibrillation; LVEF = left ventricular ejection fraction; MR = mitral regurgitation; PAF = paroxysmal atrial fibrillation; PerAF = persistent atrial fibrillation; SGLT2 = sodium-glucose cotransporter 2; SR = sinus rhythm; TIA = transient ischemic attack; TTE = transthoracic echocardiography; VHD = valvular heart disease.

∗ non-PAF = persistent or long-standing persistent atrial fibrillation.

### CCTA-based morphologic assessment of the PVs, LAPW, and LAA

Preprocedural CCTA findings are summarized in Table 2, and representative examples are shown in Figures 1 and 2. No significant differences were observed between the Rec and Non-rec groups in the ostial diameters of the left superior PV (19.2 ± 3.7 mm vs 18.8 ± 3.0 mm; *P* = .454), left inferior PV (16.4 ± 2.8 mm vs 15.8 ± 2.2 mm; *P* = .115), right superior PV (18.0 ± 4.3 mm vs 17.7 ± 2.7 mm; *P* = .537), and right inferior PV (16.8 ± 3.3 mm vs 16.1 ± 2.9 mm; *P* = .151).

**Table 2:** Preprocedural CCTA data

Variable	Recurrence (n = 63)	Non-recurrence (n = 189)	*P*
LSPV ostial diameter (mm)	19.2 ± 3.7	18.8 ± 3.0	.454
LIPV ostial diameter (mm)	16.4 ± 2.8	15.8 ± 2.2	.115
RSPV ostial diameter (mm)	18.0 ± 4.3	17.7 ± 2.7	.537
RIPV ostial diameter (mm)	16.8 ± 3.3	16.1 ± 2.9	.151
LMPV	0 (0.0)	0 (0.0)	>.99
RMPV	6 (9.5)	16 (8.5)	.797
RMPV ostial diameter (mm)	9.0 ± 1.9	9.2 ± 2.5	.820
LCPT	4 (6.3)	20 (10.6)	.322
LCPT ostial diameter (mm)	32.3 ± 4.1	27.4 ± 4.0	.091
RCPT	0 (0.0)	0 (0.0)	>.99
LA roof angle (deg)	138.1 ± 13.6	136.4 ± 12.6	.379
LA bottom angle (deg)	136.2 ± 12.8	137.5 ± 12.6	.486
LA roof line length (mm)	50.8 ± 5.4	47.4 ± 6.4	<.001
LA bottom line length, mm	51.9 ± 5.4	48.8 ± 5.7	<.001
LA posterior wall circumference (mm)	208.5 ± 12.6	193.2 ± 19.5	<.001
LA posterior wall area (mm^2^)	1989.8 ± 215.7	1769.3 ± 301.0	<.001
LAPC/LA volume index	6.0 ± 2.2	6.4 ± 1.9	.222
LAPA/LA volume index	56.8 ± 21.1	58.3 ± 18.1	.618
LAA morphology			
Cactus	10 (15.9)	31 (16.4)	.922
Chicken wing	39 (61.9)	119 (63.0)	.880
Windsock	12 (19.0)	36 (19.0)	>.99
Cauliflower	2 (3.2)	3 (1.6)	.434

Values are presented as mean ± standard deviation or n (%).

CCTA = cardiac computed tomography angiography; LA = left atrial; LAA = left atrial appendage; LAPA = left atrial posterior wall area; LAPC = left atrial posterior wall circumference; LCPT = left common pulmonary trunk; LIPV = left inferior pulmonary vein; LMPV = left middle pulmonary vein; LSPV = left superior pulmonary vein; RCPT = right common pulmonary trunk; RIPV = right inferior pulmonary vein; RMPV = right middle pulmonary vein; RSPV = right superior pulmonary vein.

Anatomical variations of the PVs, including the right middle PV (RMPV) and the left common pulmonary trunk (LCPT), were similarly observed in both groups (RMPV: 9.5% vs 8.5%; *P* = 0.797 and LCPT: 6.3% vs 10.6%; *P* = .322). The ostial diameters of the RMPV (9.0 ± 1.9 mm vs 9.2 ± 2.5 mm; *P* = .820) and LCPT (32.3 ± 4.1 mm vs 27.4 ± 4.0 mm; *P* = .091) also did not differ significantly, and no patients exhibited a right common pulmonary trunk. Morphometric assessment of the LA revealed significantly greater values in the Rec group than in the Non-rec group for the LA roof line length (50.8 ± 5.4 mm vs 47.4 ± 6.4 mm; *P* < .001), LA bottom line length (51.9 ± 5.4 mm vs 48.8 ± 5.7 mm; *P* < .001), LAPC (208.5 ± 12.6 mm vs 193.2 ± 19.5 mm; *P* < .001), and LAPA (1989.8 ± 215.7 mm^2^ vs 1769.3 ± 301.0 mm^2^; *P* < .001). In contrast, there were no significant differences in the LA roof angle (138.1° ± 13.6° vs 136.4° ± 12.6°; *P* = .379) or bottom angle (136.2° ± 12.8° vs 137.5° ± 12.6°; *P* = .486). There were also no significant differences in LAPC normalized to TTE-derived LAVI (LAPC/LAVI) or in LAPA/LAVI between the Rec and Non-rec groups. On the basis of established classification criteria,^19^ LAA morphology was categorized as cactus, chicken wing, windsock, or cauliflower, with no significant intergroup difference in distribution (cactus: 15.9% vs 16.4%; *P* = .922, chicken wing: 61.9% vs 63.0%; *P* = .880, windsock: 19.0% vs 19.0%; *P* > .99, and cauliflower: 3.2% vs 1.6%; *P* = .434). The interobserver reproducibility of LAPC measurement was good, with an ICC of 0.82 (95% confidence interval [CI] 0.66–0.91), indicating good interobserver agreement. Bland-Altman analysis showed a mean bias of –0.7 mm, with 95% limits of agreement from –20.1 to 18.6 mm (Supplemental Figure 1).

### Procedural results

Procedural characteristics are summarized in Table 3. Acute PVI was successfully achieved using CB ablation in all patients (100% in both groups), with no requirement for additional touch-up RF ablation. The mean number of CB applications required to achieve PVI was identical between the Rec and Non-rec groups (5.9 ± 1.3 vs 5.9 ± 1.8; *P* = .950). Similarly, there were no significant differences in total ablation time (16.5 ± 3.3 minutes vs 17.2 ± 4.3 minutes; *P* = .892) or minimum CB temperature (–54.5°C ± 4.3°C vs –55.5°C ± 3.5°C; *P* = .287). Cavotricuspid isthmus ablation was performed in 87 patients (34.5% overall), including 21 patients (33.3%) in the Rec group and 66 patients (34.9%) in the Non-rec group (*P* = .819).

**Table 3:** Procedural-related data and postprocedural AAD therapy

Variable	Recurrence (n = 63)	Non-recurrence (n = 189)	*P*
Total number of applications to achieve PVI	5.9 ± 1.3	5.9 ± 1.8	.950
Total ablation time to achieve PVI (min)	16.5 ± 3.3	17.2 ± 4.3	.892
Minimum CB temperature to achieve PVI (°C)	–54.5 ± 4.3	–55.5 ± 3.5	.287
CTI ablation	21 (33.3)	66 (34.9)	.819
Touch-up with an RF catheter	0 (0.0)	0 (0.0)	>.99
Acute success	63 (100)	189 (100)	>.99
Inducible ATA after PVI	21/51 (41.2)	56/166 (33.7)	.331
Procedure time (min)	80.9 ± 21.3	85.2 ± 21.0	.197
Fluoroscopy time (min)	24.4 ± 8.0	26.2 ± 10.8	.168
Adverse events			
Death	0 (0)	0 (0)	>.99
Stroke/TIA	0 (0)	0 (0)	>.99
Cardiac tamponade	0 (0)	0 (0)	>.99
Transient phrenic nerve palsy	2 (3.2)	5 (2.6)	.825
Persistent phrenic nerve palsy (>6 mo)	0 (0)	0 (0)	>.99
Gastric hypomotility	0 (0)	0 (0)	>.99
AAD therapy at discharge	35 (55.6)	91 (48.1)	.309
AAD therapy after the 3-mo BP	12 (19.0)	28 (14.8)	.426

Values are presented as mean ± standard deviation or n (%).

AAD = antiarrhythmic drug; ATA = atrial tachyarrhythmia; BP = blanking period; CB = cryoballoon; CTI = cavotricuspid isthmus; PVI = pulmonary vein isolation; RF = radiofrequency; TIA = transient ischemic attack.

Cavotricuspid isthmus ablation was indicated in patients with a documented prior diagnosis of typical atrial flutter (AFL) or with inducible ATA after PVI. ATA inducibility after PVI did not differ significantly between the groups (41.2% vs 33.7%; *P* = .331). Total procedure time and fluoroscopy time were also comparable (80.9 ± 21.3 minutes vs 85.2 ± 21.0 minutes; *P* = 0.197 and 24.4 ± 8.0 minutes vs 26.2 ± 10.8 minutes; *P* = .168, respectively).

### Complications

Procedural safety outcomes are summarized in Table 3. There were no deaths, strokes, transient ischemic attacks, cardiac tamponade, or persistent PN palsy. Transient PN palsy occurred in 7 patients overall (2.8%): 2 patients (3.2%) in the Rec group and 5 patients (2.6%) in the Non-rec group (*P* = .825). No cases of clinically significant esophageal thermal injury or symptomatic gastric hypomotility were observed.

### Follow-up

**Figure 3:**
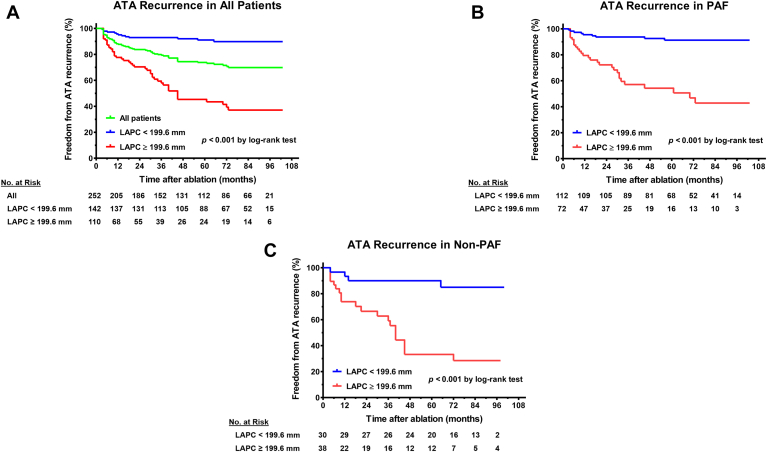
Kaplan-Meier curves for freedom from ATA recurrence after CB-PVI. Curves are shown for (**A**) all patients and the LAPC subgroups (short LAPC < 199.6 mm; long LAPC ≥ 199.6 mm), (**B**) patients with PAF stratified by LAPC (short vs long), and (**C**) patients with non-PAF (persistent or long-standing persistent AF) stratified by LAPC (short vs long). The median follow-up was 70.0 months (interquartile range 44.5–88.0 months). Numbers at risk are shown below the x-axis. *P* values were calculated using the log-rank test. AF = atrial fibrillation; ATA= atrial tachyarrhythmia; CB-PVI = cryoballoon-based pulmonary vein isolation; LAPC = left atrial posterior wall circumference; PAF = paroxysmal atrial fibrillation.

AADs were prescribed at discharge in 126 of 252 patients (50.0%), with no significant difference between the Rec and Non-rec groups (55.6% vs 48.1%; *P* = .309). After the 3-month BP, AAD therapy was continued in 78 patients (31.0%) overall (25.4% Rec vs 32.8% Non-rec; *P* = .271). All patients completed the follow-up protocol. During a median follow-up of 70.0 months (IQR 44.5–88.0 months), 63 of 252 patients (25.0%) experienced ATA recurrence: AF in 55 (87.3%) and AT in 8 (12.7%). The Kaplan-Meier estimate of freedom from ATA recurrence over the follow-up period was 69.9% (Figure 3A).

### Comparison of predictors of ATA recurrence

**Figure 4:**
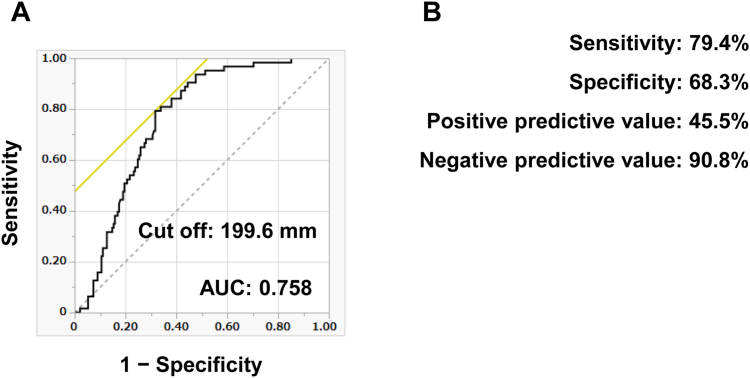
Optimal cutoff value and accuracy of the LAPC threshold for predicting ATA recurrence. **A:** ROC curve analysis (optimal cutoff value 199.6 mm; AUC 0.758). **B:** Summary of the diagnostic performance of this cutoff value, including sensitivity, specificity, positive predictive value, and negative predictive value. ATA = atrial tachyarrhythmia; AUC = area under the curve; LAPC = left atrial posterior wall circumference; ROC = receiver operating characteristic.

Results of the univariable and multivariable Cox proportional hazards analyses are summarized in Table 4. In univariable analysis, non-PAF, larger LA diameter, higher LAVI, and ≥moderate MR on TTE were associated with ATA recurrence. Preprocedural CCTA–derived anatomic parameters, including LA roof and bottom line lengths, LAPC, and LAPA, were also significant predictors. In contrast, LAPW parameters normalized to global LA size (LAPC/LAVI and LAPA/LAVI) were not associated with ATA recurrence. In multivariable analysis adjusted for clinical and echocardiographic covariates, LAPC remained the strongest regional imaging correlate of recurrence (hazard ratio 1.079 per mm; 95% CI 1.047–1.111 per mm; *P* < .001). Receiver operating characteristic curve analysis identified an LAPC threshold of 199.6 mm with the highest discriminative performance (area under the curve 0.758; 95% CI 0.698–0.818; *P* < .001) (Figure 4A), yielding a sensitivity of 79.4%, a specificity of 68.3%, a positive predictive value of 45.5%, and a negative predictive value of 90.8% (Figure 4B). Kaplan-Meier analysis demonstrated significantly higher freedom from ATA recurrence in patients with short LAPC (<199.6 mm) than in those with long LAPC (≥199.6 mm) (89.8% vs 37.1%; *P* < .001) (Figure 3A). Subgroup analyses showed consistent risk stratification across AF subtypes: in PAF, freedom from recurrence was 75.3% overall, 91.3% with short LAPC, and 42.8% with long LAPC (*P* < .001) (Figure 3B); in non-PAF, the corresponding rates were 56.4% overall, 85.0% with short LAPC, and 28.5% with long LAPC (*P* < .001) (Figure 3C). Results of the subgroup analyses are summarized in Table 5. In patients with non-PAF, all TTE parameters except LVEF, as well as all relevant CCTA-derived parameters (LA roof and bottom line lengths, LAPC, and LAPA), were significantly associated with recurrence. In contrast, in patients with PAF, only the CCTA-derived parameters were predictive whereas none of the TTE indices reached statistical significance.

**Table 4:** Predictors of ATA recurrence: univariable and multivariable Cox proportional hazards analyses

Univariable			Multivariable		
Risk factor	HR (95% CI)	*P*	Risk factor	HR (95% CI)	*P*
Baseline characteristics					
Age	0.992 (0.971–1.013)	.441			
Male sex	0.878 (0.508–1.517)	.642			
Height	1.011 (0.984–1.039)	.424			
Weight	1.008 (0.990–1.026)	.403			
BMI	1.031 (0.966–1.100)	.360			
Comorbidities					
Congestive heart failure	1.037 (0.416–2.585)	.939			
Hypertension	1.016 (0.619–1.668)	.950			
Diabetes mellitus	0.586 (0.235–1.46)	.251			
Previous stroke or TIA	0.684 (0.095–4.935)	.707			
Coronary artery disease	1.581 (0.634–3.942)	.326			
CKD (Chronic Kidney Disease Epidemiology Collaboration estimated glomerular filtration rate [CKD-EPI eGFR] < 60 mL/(min·1.73 m²))	0.406 (0.056–2.927)	.371			
Non-PAF∗	1.842 (1.112–3.051)	.018	Non-PAF∗	1.168 (0.669–2.036)	.585
Symptomatic AF episode	0.696 (0.415–1.170)	.171			
CHADS_2_ score	0.952 (0.719–1.261)	.733			
CHA_2_DS_2_-VASc score	0.960 (0.787–1.171)	.688			
HAS-BLED score	0.956 (0.724–1.263)	.753			
Preprocedural medications					
ACEI or ARB	1.181 (0.699–1.994)	.534			
Aldosterone antagonist	0.049 (0.000–13532.9)	.637			
ARNI	0.049 (0.000–289754.4)	.705			
Statin	1.093 (0.604–1.981)	.768			
β-Blockers	0.837 (0.501–1.398)	.497			
SGLT2 inhibitors	0.049 (0.000–1511.5)	.567			
Preprocedural AAD therapy					
None	0.948 (0.549–1.639)	.850			
Class Ⅰ	0.857 (0.447–1.643)	.643			
Amiodarone	1.057 (0.455–2.453)	.897			
Bepridil	1.123 (0.683–1.846)	.648			
Pharmacologic restoration of SR before the procedure in non-PAF∗	1.211 (0.632–2.321)	.565			
Pre-BNP	1.001 (0.999–1.004)	.310			
TTE parameters			TTE parameters		
LVEF	0.982 (0.959–1.007)	.158			
LA diameter	1.090 (1.041–1.140)	<.001	LA diameter	1.014 (0.948–1.085)	.680
LA volume index	1.038 (1.017–1.059)	<.001	LA volume index	1.018 (0.988–1.048)	.239
VHD (≥mild)	1.514 (0.902–2.542)	.117			
MR ≥mild	1.601 (0.897–2.860)	.112			
MR ≥moderate	2.251 (1.143–4.431)	.019	MR ≥moderate	1.308 (0.638–2.680)	.464
Preprocedural CCTA data			Preprocedural CCTA data		
LSPV ostial diameter	1.051 (0.969–1.140)	.231			
LIPV ostial diameter	1.088 (0.977–1.212)	.123			
RSPV ostial diameter	1.032 (0.945–1.126)	.487			
RIPV ostial diameter	1.064 (0.984–1.151)	.120			
RMPV	1.280 (0.552–2.969)	.566			
LCPT	0.560 (0.203–1.541)	.261			
LA roof angle	1.008 (0.988–1.027)	.454			
LA bottom angle	0.990 (0.972–1.009)	.310			
LA roof line length	1.100 (1.055–1.147)	<.001	LA roof line length	0.971 (0.909–1.037)	.380
LA bottom line length	1.088 (1.042–1.136)	<.001	LA bottom line length	0.961 (0.906–1.018)	.176
LA posterior wall circumference	1.052 (1.038–1.067)	<.001	LA posterior wall circumference	1.079 (1.047–1.111)	<.001
LA posterior wall area	1.002 (1.001–1.003)	<.001	LA posterior wall area	0.999 (0.997–1.001)	.222
LAPC/LA volume index	0.960 (0.832–1.109)	.581			
LAPA/LA volume index	1.002 (0.988–1.017)	.756			
LAA morphology					
Cactus	1.002 (0.510–1.970)	.995			
Chicken wing	0.916 (0.551–1.524)	.737			
Windsock	1.053 (0.561–1.975)	.873			
Cauliflower	1.651 (0.403–6.756)	.486			
Procedural-related data					
Total number of applications to achieve PVI	1.033 (0.870–1.228)	.710			
Total ablation time to achieve PVI	1.000 (0.998–1.001)	.757			
Minimum CB temperature to achieve PVI	1.039 (0.976–1.105)	.229			
CTI ablation	0.725 (0.411–1.280)	.267			
Inducible ATA after PVI	1.270 (0.752–2.146)	.371			
Procedure time	0.994 (0.981–1.006)	.320			
Fluoroscopy time	0.985 (0.958–1.013)	.281			
Adverse events	1.302 (0.318–5.331)	.714			
Postprocedural AAD therapy					
AAD therapy at discharge	1.107 (0.672–1.823)	.689			
AAD therapy after the 3-mo BP	1.176 (0.627–2.208)	.613			

HRs for continuous variables are per-unit increase (LA diameter per 1-mm increase, LA volume index per 1-mL/m^2^ increase, LAPC per 1-mm increase, and LAPA per 100-mm^2^ increase).

AAD = antiarrhythmic drug; ACEI = angiotensin converting-enzyme inhibitor; AF = atrial fibrillation; ARB = angiotensin receptor blocker; ARNI = angiotensin receptor-neprilysin inhibitor; ATA = atrial tachyarrhythmia; BMI = body mass index; BNP = brain natriuretic peptide; BP = blanking period; CB = cryoballoon; CCTA = cardiac computed tomography angiography; CI = confidence interval; CKD = chronic kidney disease; CTI = cavotricuspid isthmus; LA = left atrial; LAA = left atrial appendage; LAPA = left atrial posterior wall area; LAPC = left atrial posterior wall circumference; LCPT = left common pulmonary trunk; LIPV = left inferior pulmonary vein; LSPV = left superior pulmonary vein; LVEF = left ventricular ejection fraction; MR = mitral regurgitation; PAF = paroxysmal atrial fibrillation; PVI = pulmonary vein isolation; RMPV = right middle pulmonary vein; RIPV = right inferior pulmonary vein; RSPV = right superior pulmonary vein; SR = sinus rhythm; SGLT2 = sodium-glucose cotransporter 2; TIA = transient ischemic attack; TTE = transthoracic echocardiography; VHD = valvular heart disease.

∗ Non-PAF = persistent or long-standing persistent atrial fibrillation.

**Table 5:** Subgroup analyses based on AF subtype

Variable	PAF (n = 184)			Non-PAF∗ (n = 68)		
	Recurrence	Non-recurrence	*P*	Recurrence	Non-recurrence	*P*
No. of patients	38 (20.7)	146 (79.3)		25 (36.8)	43 (63.2)	
TTE parameters						
LVEF (%)	64.0 ± 9.2	65.6 ± 8.1	.335	61.5 ± 11.2	62.0 ± 8.5	.842
LA diameter (mm)	39.7 ± 5.7	38.1 ± 5.1	.107	45.1 ± 4.0	41.4 ± 5.0	.001
LA volume index (mL/m^2^)	39.7 ± 5.7	38.1 ± 5.1	.107	46.5 ± 12.2	36.7 ± 10.3	.002
VHD (≥mild)	8 (21.1)	31 (21.2)	.981	14 (56.0)	12 (27.9)	.022
MR ≥mild	4 (10.5)	21 (14.4)	.537	11 (44.0)	6 (14.0)	.006
MR ≥moderate	3 (7.9)	10 (6.8)	.823	7 (28.0)	3 (7.0)	.018
Preprocedural CCTA data						
LA roof line length (mm)	50.4 ± 5.7	47.3 ± 6.3	.004	51.3 ± 5.0	48.0 ± 6.8	.001
LA bottom line length (mm)	50.7 ± 4.9	48.4 ± 5.5	.012	53.7 ± 5.6	50.2 ± 6.4	.025
LA posterior wall circumference (mm)	207.0 ± 12.2	191.6 ± 19.2	<.001	210.6 ± 13.2	198.9 ± 19.8	.005
LA posterior wall area (mm^2^)	1959.1 ± 202.6	1750.0 ± 301.4	<.001	2036.4 ± 230.5	1836.8 ± 293.2	.003

Values are presented as mean ± standard deviation or n (%).

CCTA = cardiac computed tomography angiography; LA = left atrial; LVEF = left ventricular ejection fraction; MR = mitral regurgitation; PAF = paroxysmal atrial fibrillation; TTE = transthoracic echocardiography; VHD = valvular heart disease.

∗ Non-PAF = persistent or long-standing persistent atrial fibrillation.

### Mechanisms of recurrence and findings at second ablation procedures

Among the 63 patients with ATA recurrence, recurrent arrhythmias consisted of AF in 55 patients (87.3%) and AT in 8 patients (12.7%). Of these patients, 30 (47.6%) underwent a second ablation procedure during follow-up. Among patients undergoing a second procedure, 24 had recurrent AF and 6 had recurrent AT. Overall, PV reconnection was identified in 10 of 30 patients (33.3%) whereas 20 patients (66.7%) demonstrated durable PVI, indicating that most recurrences were not attributable to PV reconnection. Among the 24 patients with recurrent AF who underwent a second procedure, PV reconnection was identified in 8 patients whereas 16 patients had durable PVI. Among the 6 patients with recurrent AT who underwent a second procedure, PV reconnection was present in 2 patients (1 with right superior PV gap–related AT and 1 with roof-dependent AT) whereas 4 patients showed no PV reconnection. The AT mechanisms included right AT or typical AFL (n = 2), PV gap–related AT (n = 1), and roof-dependent AT (n = 3).

## Discussion

### Principal findings and interpretation

To our knowledge, this study provides novel evidence that quantitative assessment of LAPW morphology using routine preprocedural CCTA is associated with long-term ATA recurrence after CB-PVI. The extended follow-up period in this study enhances the robustness of these findings by enabling a reliable evaluation of long-term outcomes. The principal findings can be summarized as follows:

1. Enlarged LAPW geometry, characterized by increased LA roof and bottom line lengths, LAPC, and LAPA, was significantly associated with ATA recurrence.
2. In multivariable analysis, LAPC emerged as the strongest regional imaging correlate of ATA recurrence after adjustment for conventional clinical and echocardiographic predictors, including AF subtype, LA diameter, and LAVI.
3. An LAPC threshold of ≥199.6 mm provided effective risk stratification across AF subtypes in this cohort, underscoring its potential as a practical and quantifiable imaging marker.

The LAPW is a well-established arrhythmogenic substrate involved in AF initiation and maintenance because of its embryologic and electrophysiological continuity with the PVs.^8^ This region is prone to fibrotic remodeling, conduction delay, and ectopic activity.^8^ Our data indicate that geometric enlargement of the LAPW, particularly reflected by LAPC, signifies a residual arrhythmogenic substrate that persists despite successful PVI and is associated with an increased risk of long-term recurrence.^20^^,^^21^ Among the CCTA-derived parameters, LAPC may best capture the extent of LAPW remodeling because it integrates elongation along both the roof and inferior boundaries—features that may promote reentrant circuits. In contrast, LAPA, although correlated with size, is less sensitive to elongation and more affected by measurement variability, potentially explaining why LAPC remained the strongest regional imaging correlate in multivariable analysis.

### Mechanistic implications

Even in patients with PAF, enlargement of the LAPW likely reflects early structural remodeling extending beyond the PVs. In our cohort, 30 of 63 patients (47.6%) with ATA recurrence underwent a second ablation procedure. Among these patients, PV reconnection was identified in 10 (33.3%) whereas durable PVI was maintained in 20 (66.7%), indicating that most recurrences were not solely attributable to PV reconnection. Among patients with recurrent AF who underwent a second procedure, PV reconnection was observed in 8 of 24 cases whereas 16 patients demonstrated durable PVI. Among patients with recurrent AT who underwent a second procedure, PV reconnection was present in 2 of 6 cases whereas 4 patients showed no PV reconnection. The observed AT mechanisms included right AT or typical AFL, PV gap–related AT, and roof-dependent AT. These findings suggest a potential mechanistic association between LAPW enlargement and a persistent arrhythmogenic substrate beyond the PVs.

However, residual confounding from global LA remodeling cannot be excluded. Taken together, our results support the concept that increased LAPC may reflect LAPW-related substrate remodeling that persists despite successful PVI and contributes to long-term recurrence through non-PV mechanisms.

### Comparison with echocardiographic parameters

While TTE-derived indices of global LA size, including LA diameter and LAVI, were associated with ATA recurrence in univariable analysis, they lost significance after multivariable adjustment. This likely reflects collinearity, as LAPC integrates anatomical and geometric aspects of LA remodeling more comprehensively than conventional global size measures. Although LAPC correlates with overall LA enlargement, it captures a regional geometric characteristic of the LAPW rather than proportional enlargement of the LA as a whole. Consistent with this concept, normalization of LAPW parameters to LAVI attenuated their association with ATA recurrence, supporting the notion that LAPC reflects absolute regional remodeling of the LAPW rather than proportional enlargement related to global LA size. Notably, CCTA-derived parameters predicted recurrence across all AF subtypes whereas TTE indices were predictive only in non-PAF. This observation aligns with the consensus statement highlighting the expanding role of CT in electrophysiology^22^ and reinforces the value of preprocedural CCTA for detailed anatomical characterization to optimize ablation strategies.^9^^,^^23^

### Association with MR and atrial remodeling

Our findings align with emerging evidence linking atrial functional MR (AFMR) to atrial remodeling. In this study, ≥moderate MR was more frequent in patients with recurrence, and these patients also exhibited significantly larger LAPC and LAPW dimensions. Prior studies have shown that even mild AFMR accelerates atrial structural and electrical remodeling, characterized by reduced voltage and expansion of low-voltage areas, supporting a shared remodeling pathway between MR and LAPW enlargement.^24^^,^^25^

Histological and voltage-mapping data further demonstrate that early-stage AFMR is associated with reduced LAPW voltage and expansion of low-voltage areas,^26^, ^27^, ^28^, ^29^ suggesting that structural and electrical remodeling may precede clinically overt MR. These findings underscore that LAPW enlargement, whether driven by AF itself, concomitant AFMR, or both, constitutes a mechanistically plausible and prognostically significant substrate for ATA recurrence after PVI.^20^^,^^21^

### Clinical implications

From a practical standpoint, LAPC measured on routine preprocedural CCTA represents a simple and reproducible imaging marker that may help inform ablation strategies. Patients with a long LAPC may represent a subgroup for whom adjunctive ablation strategies, such as LAPW isolation (LAPWI),^30^, ^31^, ^32^ warrant further evaluation, whereas those with a short LAPC may be managed conservatively with PVI alone, minimizing procedural risks. Importantly, our procedural results confirm that CB-PVI achieved 100% acute PVI success with excellent safety and procedural efficiency, consistent with prior large-scale studies.

### Limitations

Several limitations warrant consideration. First, this was a retrospective, single-center, observational study with a modest sample size, limiting generalizability. Although multivariable adjustment mitigated confounding, residual bias cannot be excluded. Second, all procedures were performed by 2 experienced operators and all patients were treated exclusively with CB; therefore, the generalizability of our findings to other ablation technologies, such as pulse field ablation, remains to be established. Third, arrhythmia monitoring relied on scheduled Holter and symptom-triggered ECG rather than continuous or implantable monitoring, so asymptomatic or short episodes may have been missed. Fourth, AAD use persisted beyond the BP in 15.9% of patients, which could have influenced recurrence detection. Fifth, while CCTA provides excellent anatomic resolution, it lacks tissue characterization; neither late gadolinium–enhanced magnetic resonance imaging^33^^,^^34^ nor intraprocedural voltage mapping^35^^,^^36^ was performed, precluding direct assessment of fibrosis and electrical remodeling. Sixth, LAPC measurements were performed manually, introducing potential observer variability, with good interobserver agreement (ICC = 0.82). Seventh, the optimal LAPC threshold (199.6 mm) was derived internally; external validation in larger, independent cohorts is necessary. Eighth, LAPW morphology was not prospectively incorporated into ablation planning, and the clinical utility of LAPC-guided strategies remains untested. Because LAPWI was not performed at the index procedure, we cannot assess whether patients with larger LAPW morphology would derive a benefit from adjunctive LAPWI. Finally, this study focused solely on ATA recurrence as the primary outcome; the relationship between LAPW morphology and other end points, such as heart failure progression, hospitalization, or AF persistence,^37^ warrants further investigation.

### Summary

Taken together, these results provide hypothesis-generating evidence that LAPW morphology, particularly LAPC, represents a mechanistically plausible and clinically useful imaging marker for recurrence risk stratification after CB-PVI. These findings should be validated in large, prospective, multicenter studies before routine clinical adoption.

## Conclusion

Preprocedural enlargement of the LAPW, particularly an increased LAPC quantified on routine CCTA, was significantly associated with ATA recurrence after CB-PVI. LAPC represents a simple, reproducible, and quantifiable regional imaging marker that may complement conventional clinical and echocardiographic measures for preprocedural risk stratification. Although external validation is warranted, CCTA-derived LAPW metrics may help inform individualized procedural planning and follow-up strategies in patients undergoing CB-PVI.

## Disclosures

The authors have no conflicts of interest to disclose.
