# Supplementary figures and images for: Preprocedural cardiac computed tomography assessment of left atrial posterior wall morphology predicts atrial tachyarrhythmia recurrence after cryoballoon pulmonary vein isolation
Source: Heart Rhythm O2. 2026 Mar 20;7(6):1134–47. doi: 10.1016/j.hroo.2026.03.012 (PMC13307476; doi:10.1016/j.hroo.2026.03.012)

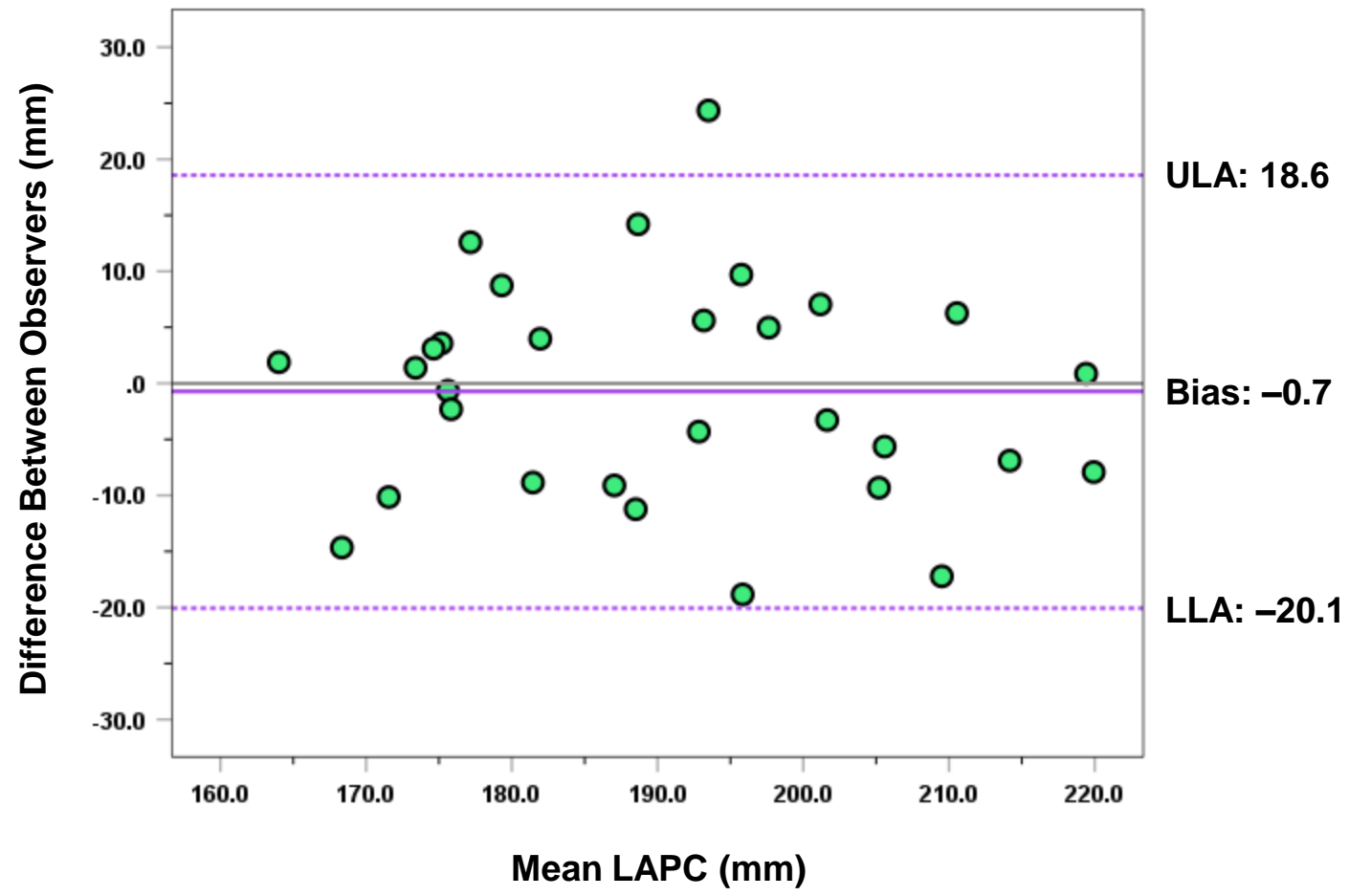

Supplementary Figure 1.

Supplement: Supplementary Figure [file mmc1.pdf]
