# Supplementary material for: Preprocedural cardiac computed tomography assessment of left atrial posterior wall morphology predicts atrial tachyarrhythmia recurrence after cryoballoon pulmonary vein isolation
Source: Heart Rhythm O2. 2026 Mar 20;7(6):1134–47. doi: 10.1016/j.hroo.2026.03.012 (PMC13307476; doi:10.1016/j.hroo.2026.03.012)
Supplement: Supplementary Material [file mmc2.docx]

**Supplementary material**

**Supplementary Figure Legend**

Supplementary Figure 1. Bland–Altman plot illustrating interobserver agreement in LAPC measurements (mm).

Abbreviations: LAPC, left atrial posterior wall circumference; ULA: upper limit of agreement; LLA: lower limit of agreement.

**Supplementary Table 1. Comparison of CCTA-derived LAPW parameters according to MR severity**

|  | None/Mild MR (n = 229) | Moderate/Severe MR (n = 23) | *p* value |
| --- | --- | --- | --- |
| The LA roof line length, mm | 48.2 ± 6.3 | 49.3 ± 7.3 | 0.030 |
| The LA bottom line length, mm | 49.3 ± 5.8 | 51.7 ± 5.7 | 0.051 |
| LA posterior wall circumference, mm | 196.3 ± 19.2 | 205.2 ± 17.9 | 0.002 |
| LA posterior wall area, mm^2^ | 1814.4 ± 292.5 | 1928.9 ± 334.8 | 0.061 |

Note: Data are mean ± standard deviation.

Abbreviations: CCTA, cardiac computed tomography angiography; LAPW; left atrial posterior wall; MR, mitral regurgitation; LA, left atrial.
